# Determinants of observing health protocols related to preventing COVID-19 in adult women: A qualitative study in Iran

**DOI:** 10.3389/fpubh.2022.969658

**Published:** 2022-08-19

**Authors:** Javad Yoosefi Lebni, Saeede Pavee, Mandana Saki, Arash Ziapour, Ahmad Ahmadi, Mehdi Khezeli

**Affiliations:** ^1^Social Determinants of Health Research Center, Lorestan University of Medical Sciences, Khorramabad, Iran; ^2^Master of Tourism Planning, Department of Geography and Tourism Planning at Kharazmi University, Tehran, Iran; ^3^Social Determinants of Health Research Center, School of Nursing and Midwifery, Lorestan University of Medical Sciences, Khorramabad, Iran; ^4^Cardiovascular Research Center, Health Institute, Imam-Ali Hospital, Kermanshah University of Medical Sciences, Kermanshah, Iran; ^5^Faculty of Psychology and Educational Sciences, Allameh Tabataba'i University, Tehran, Iran; ^6^Social Development and Health Promotion Research Center, Health Institute, Kermanshah University of Medical Sciences, Kermanshah, Iran

**Keywords:** COVID-19, women, observing the health protocols, qualitative study, adult

## Abstract

**Background:**

The best way to prevent COVID-19 is to observe health protocols. Therefore, identifying the reasons of following these protocols in order to plan and make intervention seems necessary. Therefore, the purpose of this study was to identify the determinants of observing health protocols related to prevention of COVID-19 among the Iranian adult women with a qualitative approach.

**Method:**

In this qualitative study, the conventional content analysis approach was used. saturation was obtained after face-to-face semi-structured interviews with 38 women from Kermanshah who were selected through purposeful sampling and snowball sampling. Guba and Lincoln criteria were used for the strength of the research and Graneheim and Lundman method was used for its analysis.

**Results:**

After analyzing the interviews, 5 categories, 12 subcategories and 110 initial codes were obtained. Categories and sub-categories were: 1- Individual factors (personality traits, health literacy about COVID-19); 2- Perceived risk having underlying disease in oneself and family, history of getting COVID-19 and death in close relatives; 3- Fear of the destructive consequences of the disease (concern about the economic consequences of getting the disease, concern about the treatment process); 4- Social and cultural factors (social monitoring, religious insight, ability to properly manage social interactions, impressionability from important others); 5- Environmental factors (supportive living environment, access to health and anti-infective materials).

**Conclusion:**

Increasing the adherence of adult women to health instructions related to COVID-19 requires interventions at different levels of individual, environmental and social, and without accurate knowledge of the customs and culture of a society effective interventions cannot be established.

## Introduction

COVID-19 from China spread to other parts of the world (1, 2). Due to the ambiguous nature of this virus and its high transmission power, as well as the problems it imposes on the individual and family after infection, the most important and in fact the main way to control the disease is to eliminate the virus transmission chain (3, 4). The incidence and death rate of the disease led governments to implement a set of health protocols to prevent the disease. Depending on the situation, these protocols ranged from simple health rules such as wearing a mask to quarantine and social distancing (5). Observance of issues such as the use of masks and gloves, washing and disinfecting the hands and surfaces, maintaining proper distance and using vaccines have been mentioned as ways to prevent COVID-19 (6, 7).

In Iran, on 19 February 2020, the first definitive case of COVID-19 was announced (8) and by 19 December 2021, a total of more than 6 million people have been infected with this disease, and the death rate has reached more than 131,000 (9, 10).

In line with global warnings to break the chain of transmission, the Iranian government also implemented a variety of restrictive behaviors, such as quarantining cities and closing public centers and places, and at the same time, informed the people about various methods of preventing infection through national media and social networks (11, 12). However, despite warnings from health organizations and governments, some people did not follow health protocols because of reasons such as poverty and economic hardship (13), personal characteristics, lack of access, etc. (14). Observing health guidelines has become one of the main concerns since the outbreak of COVID-19 (15, 16), which is influenced by various physical, psychological, political, social and cultural factors (17, 18). Studies of past pandemic crises, such as influenza and SARS, have shown that factors such as perceived risk of disease, strength of transmission, death rate, and stress experienced due to the disease play an important role in the type of preventive behaviors of the general public (14). The results of Webster et al.'s (19) study showed that factors such as public awareness of disease and quarantine procedures, social norms, perceived benefits of quarantine and understanding of disease risk, as well as practical issues such as resource depletion or financial consequences of unemployment are related to the degree of observing quarantine (19). In a study conducted in Indonesia, the variables of knowledge, personality and concern were reported as important determinants of observing health protocols related to COVID-19 (20). In a review study conducted by Shushtari et al. (17), living and working conditions, social support, trust, social norms, economic and social status, and mental health were reported to be the most important social determinants of following the COVID-19 health protocols. In another review study, environmental resources and contexts, belief in consequences, feelings, and social effects have been reported as important determinants of observing social distancing (21).

Regarding COVID-19, men have a higher rate of infection and death than women (22, 23), most studies have reported that health protocols observing in women is higher than men (24–27). This may be due to various reasons such as having enough opportunity, more access, more patience or also the general perception that women are more inclined to maintain their health than men (28). Of course, men's working conditions can also be a factor (14).

Given the importance of observing health protocols in the prevention of COVID-19, identifying the factors affecting the observance of these protocols is a necessity of any planning and intervention to protect public health. Most previous research has been done quantitatively and has examined the reasons for non-compliance with health protocols, and the reasons for compliance with health protocols has been less examined with a qualitative method, while a qualitative method can help a lot. Also, few studies have examined the reasons for observing health protocols among Iranian women. Since some Iranian women are illiterate, it is not appropriate to use a quantitative method to investigate this phenomenon. Therefore, this study aimed to explor the determinants of observing health protocols related to the prevention of COVID-19 in Iranian adult women.

## Materials and methods

This study was conducted with a qualitative approach and conventional content analysis method (29) among women in kermanshah. Participants in the study were women who had followed COVID-19 health protocols such as wearing masks, physical distance, washing hands, etc. over the past 2 months. Inclusion criteria consisted of being a woman, observing health protocols during the last 2 months and willingness to participate in the study. Exclusion criterion was an incomplete interview.

Purposeful sampling and then snowball sampling were used to reach the participants. Initially, a call was published in social media in order that those who follow the health protocols and are eligible to participate in the study tell their names and leave their phone numbers in order to be contacted to determine the time and place of the interview. Researchers attended the society and asked the women who were following health protocols, if they had fully observed health protocols in recent months, and if so, the interview would begin. At the end of the interview, the women were also asked to introduce other women who met the inclusion criteria among their friends and acquaintances, so that they could be contacted as soon as possible and the interview would be coordinated. A total of 16 women were selected through purposeful sampling and 22 women through snowball sampling. All interviews were recorded with the participants' consent, and field notes were used wherever the researcher needed them.

Semi-structured face-to-face interviews were used to collect data. The interview guide was developed to conduct the interviews; all the authors of the article designed questions to achieve the objectives of the research during three discussion sessions, then these questions were tested in interviews with three participants and after three pilot interviews, researchers corrected it during a meeting and the final interview was compiled (Table 1). All interview questions were asked of all participants, but their order depended on the participants' answers, and other minor questions were asked to complete the interview. The average duration of the interviews was 61 min, the shortest interview lasted 23 minutes and the longest one lasted 80 min. The time and place of the interviews were determined in advance with the participants and based on their opinions. Interviews were conducted in secluded public places such as parks or cultural sites such as libraries or the workplaces of some participants which were secluded and no one but the researcher and participant was present.

**Table 1:** The guide for interview question.

**Question**	
1	What made you observe the health protocols for preventing COVID-19?
2	Do you think that your personality had an effect on observing health protocols? Explain.
3	What do you think about health protocols and their impact on preventing COVID-19? Explain.
4	What was the most important thing that made you follow the protocols?
5	Did the words and behavior of family members, friends and other people affect your behavior? Explain.
6	Do people around you follow the health protocols like you? Explain.
7	How was your access to hygiene? Explain.

In order for the participants to be able to share their experiences more easily with the researcher, a female colleague with a master's degree in women studies who had sufficient experience in qualitative research methods and interviews was used. Data collection continued until theoretical saturation was reached. Saturation means when the continuation of the interview adds nothing to the research and the codes are repeated and no new code is formed, so the researcher decides not to continue the interviews (30, 31). In this study, saturation was obtained by interviewing 38 women.

Maxqda 2020 software was used for data classification and Graneheim and Lundman method was used for its analysis (29). Data analysis was performed by the first, second, and corresponding author of the article, with the supervision and cooperation of all authors. Thus, in the first step, immediately after each interview, the interviews were typed and saved by two members of the research team in Word 2010 software. In the second step, the text of the interviews was read and reviewed several times by the researchers to get a general understanding of the text of the interviews. In the third step, all the texts were read word for word and with great care and the codes were extracted. In the fourth step, the codes that were similar in terms of content and meaning were placed in a class and it was determined how they were related. In the fifth step, the data were placed in the main categories, which were more general and abstract than the previous classification, and the themes were extracted.

Guba and Lincoln criteria were observed to improve the quality of results (32). To increase the dependability, all contributors to the article were informed about process of analyzing and coding, and in the meetings that were held, expressed their views, and finally the names of the categories and subcategories were finalized with the approval of all authors. To increase the credibility of the study, the researchers selected participants with the greatest differences in terms of demographic characteristics to observe the principle of diversity in sampling. At the end of each interview, the researcher briefly expressed his general understanding of the participants' experiences and it should be confirmed by the participant. Also, after coding and analyzing the data, the findings of the present article were provided to 12 participants to determine whether the researchers reported their experiences correctly or not and it was confirmed after a few minor corrections. To gain Confirmability, the researchers sent data analysis and findings to 5 leading researchers in qualitative research as well as 3 people who had research experience in similar subjects to this research and, where necessary, modified them according to their opinions. In order to increase the transferability of the research, a complete description of the whole research process was provided and the quotations of the participants were given directly and in large numbers. The research findings were also sent to 6 people who met the inclusion criteria but did not participate in the study, which was finally confirmed by them.

### Ethics approval and consent to participate

To observe research ethics, the researchers considered the following issues: Ethical approval was obtained from the Kermanshah University of Medical Sciences (IR.IUMS.REC.1401.023). obtaining written consent from all participants, obtaining written consent to record the interview, introducing themselves and the necessity and objectives of the research at the beginning of each interview, observing the principles of confidentiality and maintaining the names of participants in publishing research results, determining the time and place of the interview and the desired time of cutting it by the participants, and observing the health protocols during the interview.

## Results

The present study was conducted with the participation of 38 adult women in Kermanshah, whose demographic characteristics are listed in Table 2. Findings showed that most of the participants in the age range of 30 to 50 years had higher education than diploma and were married and housewife. Also, by analyzing the data obtained from the interviews, 110 initial codes, 12 subcategories and 5 categories were obtained (Table 3).

**Table 2:** Demographic information of the participants.

**Variable**	**Group**	**Frequency**
Age	18–30	9
	30–50	18
	Over 50	11
Education	Illiterate and elementary	7
	Junior high school and diploma	12
	Higher than diploma	19
Marital status	Single	15
	Married	18
	Divorced or widowed	5
Occupation	Housewife	14
	Self-employed	10
	Employee	7
	University student	7
History of getting COVID-19 for her and her relatives	Yes	21
	No	17

**Table 3:** Codes, categories and subcategories obtained from interviews with participating women.

**Categories**	**Subcategories**	**Codes**
Individual factors	Personality traits	Responsibility, compassion, conservatism, patience, hope
	Health literacy about COVID-19	Proper knowledge of COVID-19, proper knowledge on the ways of transmitting and preventing COVID-19, proper knowledge on how to observe social distancing, proper knowledge on how to use masks and other health supplies
Perceived risk	Having underlying disease in oneself and family	Asthma, diabetes, cancer, kidney disease, heart disease and so on
	History of getting COVID-19 and death in close relatives	History of COVID-19 in family, history of COVID-19 among friends and colleagues, history of COVID-19 in neighbors and relatives
Fear of the destructive consequences of the disease	Concern about the economic consequences of getting the disease	High cost of disease treatment, high cost of medical tests, job loss
	Concern about the treatment process	Prolonged treatment, endangering other family members, disrupting her life and other family members
Social and cultural factors	Social supervision	Worry about others criticizing for not observing, worry about others distancing from them if they do not follow protocols, being stigmatized as uncultured
	Religious insight	To be responsible for one's own body, to consider endangering one's own health as a sin, to consider endangering the health of others as a sin
	Ability to properly manage social interactions	To reduce face-to-face communication and make more use of telephone communication, to use social media for communication, to use social media for social and cultural events
	Impressionability from important others	To follow the advice of celebrities, to follow the advice of religious clerics, to follow the advice of prominent doctors, to follow the advice of nurses and important people
Environmental factors	Supportive living environment	Sensitivity of other family members to observe protocols, observance of health protocols by apartment building residents, observance of health protocols by most colleagues
	Access to health and anti-infective materials	Adequate access to masks and gloves, adequate access to alcohol

### Individual factors

The first category that was obtained was individual factors consisting of two subcategories of personality traits and having health literacy about COVID-19. In fact, part of the observance of health protocols by women was related to their personality and others to their knowledge and awareness of COVID-19.

#### Personality traits

Having some personality traits in people caused them to observe health behaviors more. In this study, women who felt more responsible followed health protocols more because they considered themselves responsible for their own health and that of others. Women who were kind and compassionate also followed health protocols properly. Although observing health protocols has sometimes been difficult, women who are more patient and have more hope for the future are more likely to follow them. Also, women who are less risk-averse and have a conservative personality are more likely to follow health protocols for fear of COVID-19.

“Observing the COVID-19 health protocols is related to both myself and others. Even if I am not worried about my own health, I have to wear a mask because of the health of others, which is why I always try to follow them.” (27 years old, married with a bachelor's degree).

“I can never imagine that I could endanger anyone's health by my negligent behavior. I can't be so cruel. I think not following health protocols shows the peak of cruelty” (39 years old, married, bachelor's degree).

“I'm too patient. I've been wearing a mask since COVID-19 came. Even at home when a guest comes, I wear a mask again. Sometimes my family tells me you're so patient that you observe a lot.” (48 years old, married, under diploma).

“I love my life and I do not want to die, so I wear a mask and I do not go to crowded places” (22 years old, single with a bachelor's degree).

#### Having health literacy about COVID-19

In fact, most participants had comprehensive information on various aspects of COVID-19 and had obtained this information from a variety of sources, not just one source of information. Participants who had good information about all aspects of COVID-19, such as how to transmit and prevent COVID-19, how to observe social distance, and how to use masks and other health supplies, were more observant.

“I tried to increase my knowledge in this field from the early outbreak, so in addition to following scientific news, I collected information on various websites, and sometimes I even used English sources, so I knew what a bad disease we were facing, which made me pay more attention” (50 years old, married with under diploma education).

“My husband works in the health office and he passed on good information to us, so I tried to follow all the health protocols” (33 years old, married, higher than a diploma).

“I wear both masks and gloves and I always use alcohol. I haven't traveled since COVID-19 came because I know that not following any of these things will make me much more likely to get infected and my life and my family life will be in danger.” (51 years old, married with elementary education).

“I try not to go to crowded places at all, or every time I go I put on two masks and When I get in a taxi or bus, I immediately roll down the windows so that there is air flow, because I know that if there is no air flow, I might get infected.” (29 years old, single with a bachelor's degree).

### Perceived risk

People who feel more at risk for COVID-19 are more likely to observe health protocols. This fear increases with a history of underlying disease in the individual and family members. Also, if one of her close relatives gets COVID-19 and she observes the treatment process, she feels fearful, which causes her to follow health protocols more.

#### Having an underlying disease in herself and her family

Some participants, due to having a dangerous underlying illness in themselves or their family members, considered themselves obliged to follow health protocols. In fact, it can be said that they feel more fearful due to having an underlying disease and that made them more observant.

“I have diabetes, so I'm very scared of COVID-19 and I always try to observe because I know I will die if I get it.” (61 years old, widow, illiterate).

“My mother has asthma and her lungs are very weak. If she gets COVID-19, it is very dangerous for her, so I try to be very careful and observant so that nothing bad happens.” (37 years old, single with a diploma).

“My mother-in-law has cancer and lives with us. Because of her I have to pay a lot of attention and be very observant so that, God forbid, nothing bad happens to her” (27 years old, married with a bachelor's degree).

“My father is on dialysis and if he gets COVID-19 it is not clear what will happen to him, so I and other family members try to be very observant so that nothing bad happens” (21 years old, single with a diploma).

#### History of COVID-19 in close relatives

History of COVID-19 in close relatives and observing the life of these people after getting it and the difficulties they experienced caused the participating women to express that by observing such an experience, they feel more at risk and follow health protocols better. Also, people who experienced COVID-19 death in close relatives and family felt more at risk and observed the protocols more than others.

“My father took COVID-19, he got very bothered, our lives were disrupted for a few weeks. Frankly speaking, after my father became ill, I was very scared and I observed more” (35 years old, single with a bachelor's degree).

“At first I thought that COVID-19 was not dangerous for me, but when I saw that our neighbor had taken COVID-19 and was being bothered by it a lot, I was scared and tried to observe more.” (65 years old, divorced, illiterate).

“One or two of my friends and colleagues took COVID-19 and went to the brink of death, and one of them died. When I found out that my colleague was dead, I was very worried and I have been observing more since that day.” (44 years old, married with a bachelor's degree).

### Fear of the destructive consequences of the disease

This category on the one hand refers to the economic consequences of COVID-19 and on the other hand is concerned about the process of treatment of the disease that may endanger the health of associates and disrupt their lives.

#### Concerns about the economic consequences of getting the disease

Getting COVID-19 in Iran is associated with high economic costs, and families with a COVID-19 patient have to spend a lot of money on a daily basis. Also, with getting this disease and deprivation of work, people's income decreases, so some participants stated that they followed COVID-19 health protocols for fear of the economic consequences of it.

“If I get covd-19, I have to stay home until I die, because they say the cost is too high, so I try to observe a lot not to get infected.” (27 years old, single with a bachelor's degree).

“I am a hairdresser. If I take COVID-19, I have to stay home for at least a few weeks and my job is closed, so I have to be very observant in order not to lose my job” (42 years old, divorced with a bachelor's degree).

“I observe because I know the cost of medical tests, etc. is too high. Also, because public hospitals do not have the capacity, I have to go to a private hospital where overnight expenses are the same as my husband's a month income” (37 years old, married with a master's degree).

## Social and cultural factors

Social and cultural factors were one of the important determinants of observing health protocols by participants. Part of these social factors were related to social pressures that required a person to observe health protocols, part to the religious views of individuals about their own health and the health of others. Proper management of social interactions and being influenced by important others were other social and cultural factors.

### Social monitoring

In Iranian society, someone who does not follow health protocols, especially the use of masks, is usually reprimanded by the public, and others may distance themselves from him or criticize him for not observing health protocols properly. Most participants stated that they followed health protocols for fear of being rejected or warned by others. In fact, non-compliance with health protocols in society causes others to label the wrongdoer as a silly person, and this issue causes some people to observe health protocols to avoid such labels.

“I get very upset that someone criticizes me, so I always try to follow health protocols so that no one nags at me” (32 years old, married with a master's degree).

“If I see that someone does not follow the health protocols, I distance myself from them and do not talk to them, and since I do not want anyone to treat me like this, I try to follow the health protocols as much as I can.” (67 years old, married, illiterate).

“In the bus, anyone who does not wear a mask is labeled a thousand times, and I really give people the right. I do not want anyone to think that I do not have understanding and wisdom, so I try to wear a mask” (55 years old, married with a bachelor's degree).

“There is a friend of mine who does not believe in wearing a mask at all, etc. Other friends label her a thousand times.” (47 years old, widow, under diploma).

### Religious insight

Religious people consider themselves responsible for their own health and the health of others, and consider any harm to their own health and the health of others a sin. According to these people, non-observance of health protocols is an example of intentional harm to oneself and others because it endangers health and this is a sin, so they try to observe health protocols as much as they can in order to avoid sin.

“God has given us a body which we have to take care of, and if we are not careful we have sinned” (38 years old, married with a master's degree).

“Just as drug use is a sin and endangers health, not observing protocols is just as, and perhaps more, a sin because it endangers the health of others in addition to one's own health” (41 years old, married with a master's degree).

“If I do not follow the protocols and cause someone else to get COVID-19, I have committed a great sin, so I always try to observe it for the sake of others” (29 years old, single with a bachelor's degree).

“When I know that not wearing a mask endangers the health of others, I convince myself to wear a mask even if I am bothered, because if I cause the health of others to be endangered, I have committed a sin and I will be punished in the Hereafter” (40 years old, single, under diploma).

### Ability to properly manage social interactions

Iranian culture is based on stable social relationships, so that most Iranians visit close relatives during the week, which can be dangerous during COVID-19 prevalence. While the ability to properly manage these interactions can be very helpful, some participants tried to fill this gap by reducing face-to-face communication and making more use of telephone and virtual communication, and by holding various social events in virtual space, they were could observe health protocols.

“My main concern about not following health protocols was related to attending various events that I tried to participate in them in through virtual space as much as possible” (39 years old, married with a bachelor's degree).

“I did not attend any ceremonies from the beginning of COVID-19. I called and congratulated or offered my condolences wherever necessary.” (29 years old, single with a bachelor's degree).

“During COVID-19 period, I did not attend all the birthday parties and various anniversary ceremonies, etc. We made it through virtual space.” (44 years old, married with a bachelor's degree).

### Impressionability from important others

There are always some people in individuals' lives who play a decisive role in shaping behavior. For this reason, in the face of COVID-19, some participants stated that they had tried to follow health protocols, following the statements of some celebrities, clerics, prominent doctors, nurses and other important people in life. And perhaps in the absence of such people, the rate of compliance with health protocols would be lower than before.

“When I saw celebrities wearing masks and holding their weddings in private, I learned to follow health protocols” (44 years old, married with a bachelor's degree).

“Many of our religious scholars have told us to observe health protocols. Well, we have to follow their instructions” (42 years old, divorced, under diploma).

“Doctors have been very bothered during these 2 years. When I see they want us to observe. The least we can do is to be careful about our behavior” (35 years old, single with a doctorate).

“Many nurses have lost their lives in order to maintain our health. The only thing they ask us to do is to observe more. I try to do it because of them” (57 years old, married with a bachelor's degree).

### Environmental factors

Environmental factors consisting of two sub-categories of supportive living environment and access to health and anti-infective materials were other determinants of health protocols. In fact, if people are in an environment where most people follow the protocols and also have access to the necessary health materials, the rate of compliance with the protocols will increase, as most participants stated that most people in their environment were sensitive to health protocols and they have had sufficient access to hygienic materials such as glove, masks, etc., and this has led to their encouragement to observe health protocols.

### Supportive living environment

Most of the participants stated that other family members followed the health protocols and were sensitive to their behavior, which led them to observe more. Also, some people considered the observance of health protocols by colleagues and neighbors as the reason for their observance.

“My mother reminds me every day to put on a mask and wear gloves when I go out, which makes me very self-conscious” (37 years old, single, with PhD degree).

“In our apartment building, from the early time the COVID-19 came, we took a washing liquid and put it in the yard, and we required all people to wash their hands with water and liquid when they come from the outside, and they do not have the right to take off their masks until they reach their apartment. These made me become more sensitive to the observance of health protocols” (62 years old, married with a diploma).

“Many families do not care and they go to party every day, but our family is not like that. We have observed a lot during these 2 years. When I see that we observe that much at home, I also try to observe it outside as much as I can.” (27 years old, married with a bachelor's degree).

“Most of my colleagues follow the protocols, and when I see them, I observe. If they did not observe them, I would not observe them.” (44 years old, married with a bachelor's degree).

### Access to hygienic and anti-infective materials

Adequate access to hygienic materials causes other people not to use the lack of materials as an excuse not to observe health protocols, and this issue itself can affect the observance of health protocols.

“We have several packages of masks in different designs and colors at home. I have no problem accessing the masks.” (32 years old, married with a master's degree).

“I have both hand sanitizer and surface sanitizer for each of my children. I check every time they go out to take them.” (39 years old, married with a bachelor's degree).

“I always put a mask and alcohol in my bag to use whenever I need.” (27 years old, single with a bachelor's degree).

## Discussion

The aim of this study was to explor the determinants of observing health protocols related to the prevention of COVID-19 in Iranian adult women with a qualitative approach. The results showed that the observance of health protocols by women is affected by various factors, some of which are related to the personality of individuals and others to the social factors and behaviors of others and the social environment in which they live.

Individual factors consisting of personality traits and having health literacy about COVID-19 was one of the important determinants of observing health protocols by women. In fact, women who felt more responsible for their own health and that of others around them were more likely to follow health protocols. In Ningsih et al. (33) study, a significant relationship was found between social responsibility and observance of health protocols. Also, due to the dangerous conditions of COVID-19, women who had a conservative personality followed health protocols better to avoid getting COVID-19. And because observing health protocols was sometimes really difficult and tedious, women who were patient were more inclined to follow health protocols. This finding is consistent with the research of Soleimanvandi Azar et al. (14) conducted in Iran because in their research, laziness and impatience were reported as one of the most important reasons for not wearing masks and observing other health protocols. In the research of Sari and Fawzi (20) personality traits have also been reported as one of the important determinants of compliance with health protocols.

Having a health literacy about COVID-19 was another determinant of women's observance of health protocols. In most studies, health literacy in the field of COVID-19 has been reported as one of the most important reasons for compliance or non-compliance with health protocols (34–36). In fact, the more people know about prevention methods and how to follow health protocols, the more they are encouraged to follow these protocols.

Perceived risk, consisting of two subcategories of underlying disease in oneself and family, as well as a history of COVID-19 and death in close relatives, was one of important determinants of women's observing health protocols. This finding was in line with the 2021 Plohl and Musil study (37). Also in Wise et al. (38) research, understanding the risk and understanding the economic and health effects of COVID-19 have been reported as factors affecting the observance of COVID-19 health protocols. Because the incidence and death from COVID-19 were higher in people with the underlying disease, women who had such diseases or a family member of theirs had, felt more at risk and tried to follow health protocols. They also felt more at risk when they saw the conditions of people with COVID-19 or their death, and tried to observe health protocols. In a study conducted in Italy, susceptibility of risk of contracting COVID-19 disease was significantly associated with observing health protocols related to the prevention of COVID-19 (25).

Another important finding of the study was the fear of the destructive consequences of COVID-19. In the study of Harper et al. (39), fear and anxiety about COVID-19 played an important role in influencing hygienic behaviors such as hand washing and social distancing. The expenses of COVID-19 patients in Iran were sometimes very high due to shortages of medical supplies, and the families of the patients had to pay a lot of money, and the disease itself caused people to stay away from working conditions for weeks or even months. This issue put a lot of economic pressure on the family, therefore, COVID-19 in Iran would lead to a lot of economic pressure on families, so some participants observed protocols to prevent these costs.

Concern about the disease treatment process was another determinant of women's adherence to health protocols. In fact, in addition to the economic cost of COVID-19, the length of the treatment process and the risk to other family members and disruption of their lives caused women to observe health protocols. In the study of Webster et al. (19), understanding the risk of disease has been reported as one of the important determinants of adhering to health protocols.

Social and cultural factors were another new and interesting finding in this study and showed that the health behavior of individuals is affected by the cultural and social context of societies and to intervene more effectively in this field, cultural and social components must be considered. In the study of Indrayathi et al. (40), social norms have been mentioned as one of the determinants of compliance with health protocols. In most researches on the observance or non-observance of health protocols related to COVID-19 social and cultural factors have been reported as one of the important determinants (17, 41).

Social monitoring was one of the important determinants of women's adherence to health protocols, which was one of the new and significant findings in this study. When most people in the environment follow health protocols and a person ignores these protocols, it causes other people in the community to warn that person and look at him negatively and stigmatize them and show with their behaviors that they would not like to communicate with them. In fact, it can be said that by turning health behaviors into a social norm, people accept it more easily and there is no need for government inspectors to implement it, but it is inspected and controlled by the people themselves (18). Therefore, it seems that in order to observe health protocols more, people can be encouraged more by creating more social supervision, rather than legislation and legal punishment.

Religious insight was another important determinant of health protocols. Religious people consider themselves responsible for their own health and even that of others, and according to the rules of Islam, which is the most common religion in Iran, harming their own health and the health of others is forbidden and punishable. Therefore, most women considered not following health protocols as harming their own health and the health of others, so they tried to follow these protocols as much as possible. In general, various studies have shown that during the COVID-19 pandemic, the number of religious practices increased and most people prayed or performed religious acts to end COVID-19 (42). Yoosefi lebni et al. 2021 in a study conducted among housewives in Iran during the COVID-19 period reported that women resorted to religious practices such as praying, supplication, etc. to relieve the anxiety caused by the COVID-19 outbreak (43). However, in some communities, religious people may describe quarantine laws or other health protocols as anti-religious because they stay them away from mass religious practices, so they may not follow these protocols (44). Of course, due to the religious context of Iranian society, clerics were also effective in observing health protocols because they invited people from different forums to observe health protocols (45).

Another new and interesting finding in this study was the ability to properly manage social interactions, which was one of the important determinants of observing health protocols. In fact, this finding showed that even in communities where there are a lot of family interactions, COVID-19 expansion can be prevented with proper management of these interactions. Expanding the use of social media for communication can be a good alternative to real communication, so it is suggested that health officials and planners facilitate and accelerate the use of virtual communication in society. In societies like Iran, where people still have extensive contact with close and distant relatives, observing health protocols such as social distancing and quarantine had become a problem, and the cases of COVID-19 increased whenever there were special social ceremonies. In a study conducted by Soleimanvandi Azar et al. (14). In Iran, they reported that social customs are one of the main obstacles to compliance with health protocols in Iran. In another study conducted among Iranian women, the use of social media was reported as one of the alternative solutions for real communication in the COVID-19 period (43).

Impressionability from important others was another important finding in this study. In any society, some people learn by looking at the lifestyles of those who are important to them or try to behave like them. Now these important others may come from every stratum and guild in society (such as artists, clerics, footballers, prominent doctors, Etc.). Various studies have reported the effect of celebrities in encouraging people to follow COVID-19 protocols (46, 47).

Environmental factors including a supportive living environment and access to health and anti-infective materials was another determinant of observing health protocols. When in a community in living and working places, most people follow health protocols and are sensitive to the behavior of others and feel responsible, other people in that community are encouraged to follow health protocols. Research by Coroiu et al. (26) also showed that people's health behaviors to prevent COVID-19 are influenced by the behavior of other people living in the community, that is, when people see that most people observe health protocols, they are encouraged to observe the protocols.

Access to sanitary and anti-infective materials was another determinant of compliance with health protocols. In most previous researches, lack of access or difficult access to health and anti-infective materials has been reported as one of the main reasons for not observing health protocols (14, 48, 49).

### Strengths and limitations of the study

This study is one of the few studies that qualitatively seeks to identify the determinants of observing health protocols related to COVID-19 among Iranian adult women, which can provide new and first-class information to health and social planners so that they can intervene to increase people's adherence to health protocols. There were some limitations in this study. One of the main limitations was that some participants were reluctant to do the interview due to fear of getting COVID-19. The researchers obtained their consent by explaining the conditions of the interview and the full observance of health protocols such as the use of masks, gloves, etc. during the interview. The willingness of the participants as well as the observance of some social customs caused the researchers to use a trained and experienced woman in the field of interviews and qualitative research to conduct interviews.

## Conclusion

The results showed that women observe health protocols under the influence of various factors such as individual factors, perceived risk, fear of the destructive consequences of the disease, social and cultural factors and environmental factors. Therefore, increasing the adherence of adult women to the health instructions related to COVID-19 requires interventions at different levels. At the individual level, it seems necessary to promote health literacy about COVID-19, to strengthen their sense of responsibility for their own health and the health of others, to increase the feeling of fear of getting COVID-19, and to show the consequences of getting it in the media, in order to encourage people to follow protocols more. At the social level, strengthening and cultivating more social monitoring to control the behavior of people who do not follow health protocols, using the capacity of religious clerics and celebrities to encourage people to observe health protocols, and training and instructing to better manage social interactions are seemed necessary. At the environmental level, it appears necessary to provide and make available health supplies to the public so that they can observe health protocols without any worries or restrictions.

## Data availability statement

The original contributions presented in the study are included in the article/supplementary materials, further inquiries can be directed to the corresponding authors.

## Ethics statement

The studies involving human participants were reviewed and approved by the Kermanshah University of Medical Sciences (IR.IUMS.REC.1401.023). The patients/participants provided their written informed consent to participate in this study.

## Author contributions

JY, MK, and AA were responsible for the study conceptualization and led the paper's writing. JY and AZ conducted the literature review and assisted in writing the paper. SP and AA performed the analysis, assisted in interpreting the data, and writing the paper. JY and MS assisted with the interpretation of the results and drafting programmatic implications and responsible for the data collection and coordination of the study. AZ co-led the conceptualization, supervised all aspects of writing the paper, and provided extensive comments on the manuscript. All authors were responsible for the study. All authors have read and approved the final manuscript.

## Funding

This study received funding from Kermanshah University of Medical Sciences (4010279). The funder was not involved in the study design, collection, analysis, interpretation of data, the writing of this article or the decision to submit it for publication.

## Conflict of interest

The authors declare that the research was conducted in the absence of any commercial or financial relationships that could be construed as a potential conflict of interest.

## Publisher's note

All claims expressed in this article are solely those of the authors and do not necessarily represent those of their affiliated organizations, or those of the publisher, the editors and the reviewers. Any product that may be evaluated in this article, or claim that may be made by its manufacturer, is not guaranteed or endorsed by the publisher.
